# The Pleiotropic Regulator AdpA Regulates the Removal of Excessive Sulfane Sulfur in *Streptomyces coelicolor*

**DOI:** 10.3390/antiox12020312

**Published:** 2023-01-29

**Authors:** Ting Lu, Qingda Wang, Qun Cao, Yongzhen Xia, Luying Xun, Huaiwei Liu

**Affiliations:** 1 State Key Laboratory of Microbial Technology, Shandong University, 72 Binhai Road, Qingdao 266237, China; 2 School of Health and Life Sciences, University of Health and Rehabilitation Sciences, Qingdao 266071, China; 3 School of Molecular Biosciences, Washington State University, Pullman, WA 991647520, USA

**Keywords:** zero-valent sulfur, reactive sulfane sulfur, thioredoxin, AdpA, *Streptomyces*

## Abstract

Reactive sulfane sulfur (RSS), including persulfide, polysulfide, and elemental sulfur (S_8_), has important physiological functions, such as resisting antibiotics in *Pseudomonas aeruginosa* and *Escherichia coli* and regulating secondary metabolites production in *Streptomyces* spp. However, at excessive levels it is toxic. *Streptomyces* cells may use known enzymes to remove extra sulfane sulfur, and an unknown regulator is involved in the regulation of these enzymes. AdpA is a multi-functional transcriptional regulator universally present in *Streptomyces* spp. Herein, we report that AdpA was essential for *Streptomyces coelicolor* survival when facing external RSS stress. AdpA deletion also resulted in intracellular RSS accumulation. Thioredoxins and thioredoxin reductases were responsible for anti-RSS stress via reducing RSS to gaseous hydrogen sulfide (H_2_S). AdpA directly activated the expression of these enzymes at the presence of excess RSS. Since AdpA and thioredoxin systems are widely present in *Streptomyces*, this finding unveiled a new mechanism of anti-RSS stress by these bacteria.

## 1. Introduction

The physiological functions of hydrogen sulfide (H_2_S) have been studied for about two decades and was initiated by the discovery of endogenously generated H_2_S in mammalian tissue [1]. Currently, the conception that H_2_S is the third “gasotransmitter” has been widely accepted because it is involved in numerous physiology and biological processes [2,3,4,5,6]. H_2_S acts as a signaling molecule mainly via two mechanisms. One involves the interaction and modification of the cysteine residues (persulfidation) of some target hemoproteins or proteins by H_2_S [7,8]. Concerning the other mechanism, numerous studies indicate that the direct effector is zero-valent sulfur (S^0^), the oxidation product of H_2_S. Inside cells, S^0^ exists in many forms, including persulfide (HSSH and RSSH) and polysulfide (HS_n_H and RS_n_H, n > 2) [9,10,11], which are collectively named as reactive sulfane sulfur (RSS).

The endogenously produced RSS has many beneficial functions, such as quenching the strong destructive effect of hydroxyl radical on lipid peroxidation [12,13], contributing to the de novo biosynthesis of cysteine [14], and antagonizing electrophilic stress caused by heavy metal ions [15,16], but it has harmful effects at high intracellular concentrations. The octasulfur (S_8_) has been used as a common bactericide and fungicide for many years [17,18]. A recent study indicated that S_8_ depletes glutathione (GSH) and causes disulfide stress in the yeast *Saccharomyces cerevisiae* [19]. Consequently, cells need to maintain intracellular RSS homeostasis. Persulfide dioxygenase (PDO) plays an important role to remove excess RSS in most microorganisms [20,21]. It oxidizes RSS to sulfite (SO_3_^2−^) using oxygen (O_2_) as a co-substrate. It is unclear whether there is other mechanism(s) involved in antagonizing RSS stress.

*Streptomyces* spp. are filamentous Gram-positive bacteria colonizing a wide range of terrestrial and aquatic niches [22,23,24,25]. AdpA is a transcriptional regulator that is universally present in *Streptomyces* spp. [26,27,28]. It is a key regulator of both secondary metabolism and morphological differentiation [29,30,31,32]. We previously demonstrated that AdpA senses intracellular RSS via the persulfidation of its Cys residues and affects actinorhodin production and morphological differentiation in *Streptomyces coelicolor* [33]. Herein, we report that AdpA was indispensable for maintaining intracellular RSS homeostasis. It activated expression of thioredoxins, thereby reducing excess RSS to H_2_S that may evaporate into the gas phase or be consumed by other microorganisms living in the same habitat. This finding expands the function spectrum of the versatile regulator AdpA.

## 2. Materials and Methods

### 2.1. Strains, Plasmids, and Growth Conditions

All strains and plasmids used in this study are summarized in Supplementary File (Table S1). The wild type *S. coelicolor* strain and its derivations were cultivated on mannitol soya flour (MS) solid medium [34] for spore production and intergeneric conjugation. Liquid YBP (yeast–beef–peptone) medium [35] was used for cultivation when RNA extraction, growth analysis, H_2_S measurement, intracellular RSS quantitation were performed. Minimal medium (MM) [36] and N-Evans media (2 mM Na_2_SO_4_, 1 µM Na_2_MoO_4_, 1.25 mM MgCl_2_, 10 mM KCl, 0.25 mM CaCl_2_, 2 mM citric acid, 25 mM TES, 0.5% Evans trace elements, 2.5% glucose, 100 mM NaNO_3_, 2 mM NaH_2_PO_4_, and pH 7.2) were used for detecting the sensitivity of strains to sulfur stress [37]. *Escherichia coli* DH5α was used for plasmid construction and *Escherichia coli* BL21 was used for AdpA expression. *Escherichia coli* ET12567 (pUZ8002) was used to transfer nonmethylated DNA into *S. coelicolor* M145. *S. coelicolor* strains grew at 30 °C. *E. coli* strains grew at 37 °C.

### 2.2. S. coelicolor ΔadpA, ΔadpA::adpA, and Trx-Overexpression Strains

All primers used in this study are listed in Supplementary File (Table S2). To overexpress *trx* genes, the coding sequence of these genes were amplified by PCR using the corresponding primers. The amplicon was purified and inserted into pre-cut integrating plasmid pMS82 (containing the kasOp* promoter [38]) to generate pCom-trxA, pCom-trxA3/C, pCom-trxA4, and pCom-trxB. These plasmids were introduced into *S. coelicolor* M145 using a conjugation method [39]. The obtained strains were named as wt::*trxA*, wt::*trxA3/C*, wt::*trxA4*, and wt::*trxB*.

### 2.3. Quantitative Determination of H_2_S and RSS Content

The released H_2_S from cultures was measured with a lead–acetate paper strip method [40]. Specifically, *S. coelicolor* and its derivations (2 × 10^6^ spores) were cultured in liquid YBP medium. The lead–-acetate paper strip was placed in the gas phase and replaced every 24 h. For cellular RSS determination, a previously reported method was used [41]. Briefly, 1-OD_450_ cells were collected by centrifugation (12,000× *g*, 5 min, 4 °C) and resuspended in 220 µL of Tris-HCl buffer (50 mM, pH 9.5, 1% Triton, 50 µM DTPA). 200 µM DTT or 1 mM sulfite was added as required. The cells were broken up at 95 °C for 10 min to release RSS, which then reacts with sulfite to form thiosulfate. After centrifugation at 12,000× *g* for 3 min, 50 µL supernatant was mixed with 5 µL mBBr (50 mM) for derivatization in the dark for 30 min, and then 110 µL acetic acid and acetonitrile mixture (*v*/*v*, 1:9) was added to stop the reaction. Finally, the samples were analyzed using HPLC with a fluorescence detector.

### 2.4. RNA Preparation and RT-qPCR

To extract RNA, *S. coelicolor* M145 and its derivations were grown at 30 °C in liquid YBP medium, and the mycelia were collected. Total RNA was isolated with a SteadyPure universal RNA extraction kit (Accurate Biology, Changsha, China) and treated twice with Turbo DNA-free DNase reagents (Ambion, Austin, TX, USA). The quality of RNA was analyzed by electrophoresis. RT-PCR experiments were conducted with a reverse transcriptase kit (Accurate Biology, Changsha, China), and RT-qPCR assay was performed on a Roche LightCycler480 thermal cycler system as previously described [42], using the primers listed in Supplementary File (Table S2). The data were normalized to the expression level of the *hrdB* gene that encodes the major sigma factor of *Streptomyces* [43].

### 2.5. AdpA Expression and Purification

The *adpA* coding sequence was amplified from genomic DNA using primers adpA-exp-f (with an *Nde*I adaptor) and adpA-exp-r (with an *Xho*I adaptor) (Table S2), and the PCR product was purified and inserted into *Nde*I/*Xho*I-cut pET15b to generate pEX-*adpA*. After sequence verification, the plasmid was transformed into *E. coli* BL21(DE3) cells. The cells were grown in LB medium at 37 °C to an optical density at 0.6 (OD_600_ nm), induced by adding 0.4 mM isopropyl β-d-1-thiogalactopyranoside (IPTG), and further incubated at 16 °C overnight. The cells were collected by centrifugation and washed twice with ice-cold lysis buffer (50 mM NaH_2_PO_4_, 300 mM NaCl, and 20 mM imidazole, pH 8.0). Next, these cultures were resuspended in the lysis buffer and disrupted though a SPCH-18 pressure cell homogenizer (Stansted Fluid Power LTD, Harlow, United Kingdom). The lysate was centrifuged at 5000× *g* for 10 min, and the supernatant was loaded onto Ni-NTA-Sefinose column (Sangon, Shanghai, China) with the binding buffer (50 mM NaH_2_PO_4_, 250 mM NaCl, 20 mM imidazole, pH 8.0), the washing buffer (50 mM NaH_2_PO_4_, 250 mM NaCl, 40 mM imidazole, pH 8.0), and the elution buffer (50 mM NaH_2_PO_4_, 250 mM NaCl, 250 mM imidazole, pH 8.0). The eluted protein was centrifuged in ultrafiltration tubes (Millipore), and then loaded onto a PD-10 desalting column (GE Healthcare) for buffer exchange to a sodium phosphate buffer (50 mM NaH_2_PO_4_, 50 mM NaCl and 10% glycerin, pH 8.0). The isolation process was performed at 4 °C. The purity of the protein was assessed by SDS-PAGE, and its concentration was determined by using the bicinchoninic acid (BCA) protein assay reagent (Thermo Fisher Scientific, Waltham, MA, USA).

### 2.6. Electrophoretic Mobility Shift Assays (EMSA)

EMSA experiments were performed as previously described [33]. Briefly, DNA probes containing the upstream region of selected genes were amplified by PCR with primers listed in Supplementary File (Table S2). The PCR products were purified using a gel extraction kit (Omega Bio Tek, Guangzhou, China), and the probe concentrations were determined by using a Thermo NanoDrop. The 20 nM probes were mixed with different amounts of purified AdpA in the EMSA binding buffer (20 mM Tris-HCl, 2 mM EDTA, 20 mM KCl, 0.5 mM DTT, 4% Ficoll-400, pH 8.0) containing 2 µg poly(dI-dC) at room temperature for 25 min. After incubation, samples were loaded and separated on 8% (*w*/*v*) non-denaturing polyacrylamide gels in ice-cold 0.5% Tris-Boric acid-EDTA at 120 V, and the images were captured with a Flour ChemQ system (Alpha Innotech, San Leandro, CA, USA).

## 3. Results

### 3.1. Deleting AdpA Increased the Sensitivity of S. coelicolor M145 to RSS

**Figure 1:**
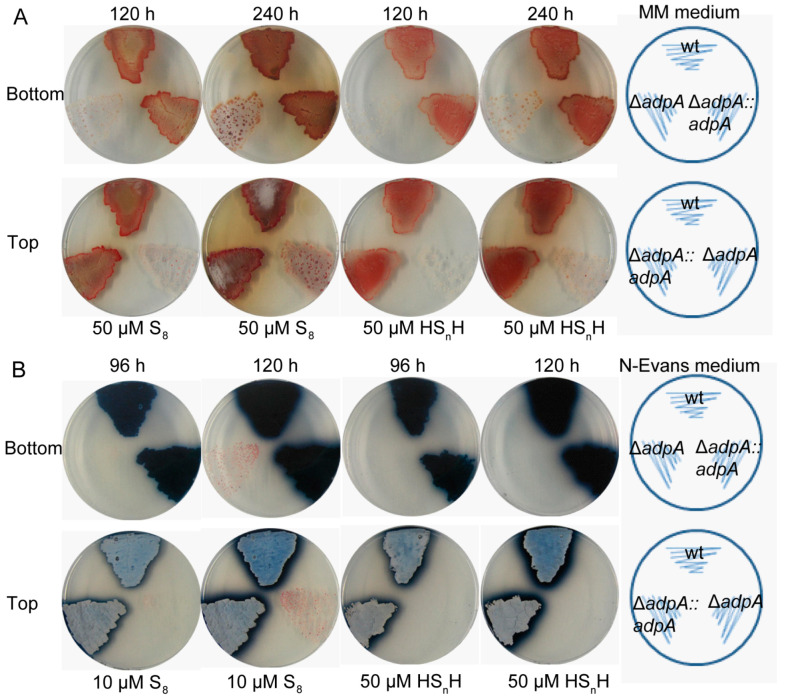
The Δ*adpA* strain was more sensitive to RSS than wt. (**A**) Phenotypes of *S. coelicolor* M145 (wt), Δ*adpA,* and Δ*adpA::adpA* grown at 30 °C on MM agar containing 50 µM S_8_ or 50 µM HS_n_H. The red pigment was undecyl prodigiosin in the colonies. (**B**) Phenotypes of wt, Δ*adpA,* and Δ*adpA::adpA* grown on N-Evans agar plates containing 10 µM S_8_ or 50 µM HS_n_H. The blue pigment is actinorhodin in the colonies. Images were taken at 120 h and 240 h (panel (**A**)) or 96 h and 120 h (panel (**B**)), and the views from top and bottom sides of the plates.

On MM plates with 50 µM HS_n_H or 50 µM S_8_, the *adpA* (*sco2792*, Gene ID: 1098226) deletion strain (Δ*adpA*) displayed severely impaired growth in comparison with the wild type strain (wt), and the growth was restored by *adpA* complementation (Δ*adpA::adpA*) (Figure 1A). For confirmation, we conducted the same experiments with another medium, the chemically limited medium (N-Evans medium), in which 50 µM HS_n_H or 10 µM S_8_ was added. Again, the growth of Δ*adpA* was obviously impaired and the growth of Δ*adpA::adpA* was fully recovered (Figure 1B). The growth of the strains was also reflected through the production of two secondary metabolites: blue-pigmented actinorhodin and red-pigmented undecylprodigiosin. *S. coelicolor* M145 produces blue-pigmented actinorhodin in MM medium [44], but red-pigmented undecylprodigiosin in N-Evans medium [45].

### 3.2. Deleting AdpA Led to RSS Accumulation in S. coelicolor M145 Cells

**Figure 2:**
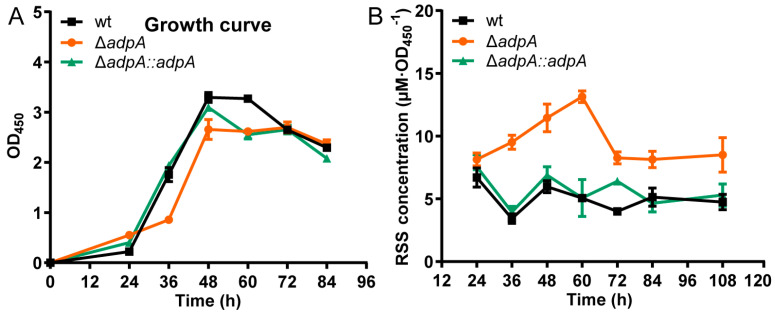
The accumulation of RSS content in Δ*adpA*. (**A**) The growth curve of *S. coelicolor* M145 wt (black bar), Δ*adpA* (red bar), and Δ*adpA::adpA* (green bar) cultured in YBP medium. OD_450_ was checked to represent the growth. (**B**) Deletion of *adpA* increased intracellular RSS. RSS concentrations of wt (black bar), Δ*adpA* (red bar), and Δ*adpA::adpA* (green bar) were detected at the indicated times.

To investigate whether AdpA affects RSS metabolism in *S. coelicolor* M145, we cultivated wt, Δ*adpA*, and Δ*adpA::adpA* in YBP medium. Compared with wt and Δ*adpA::adpA*, Δ*adpA* showed a slightly impaired growth (Figure 2A). We then analyzed the intracellular RSS contents of *S. coelicolor* M145. Δ*adpA* accumulated higher content of intracellular RSS than wt and Δ*adpA::adpA*. For Δ*adpA*, the maximum content reached 13.13 µM·OD_450_^−1^ (60 h), but in comparison, wt and Δ*adpA::adpA* had the maximum content lower than 6.70 µM·OD_450_^−1^ (Figure 2B).

### 3.3. AdpA-Dependent Expression of Thioredoxins Was Induced by RSS

**Figure 3:**
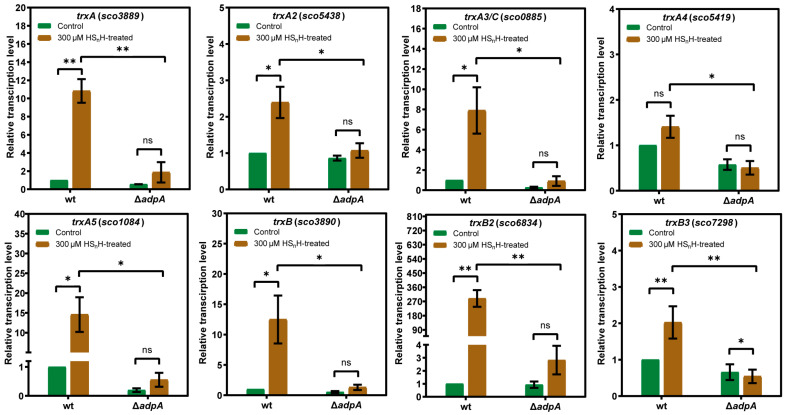
The *trx* genes in *S. coelicolor* M145 were induced in response to HS_n_H treatment in an *adpA*-dependent manner. RT-qPCR analysis was performed to compare the transcriptional level of *trxA*, *trxA2*, *trxA3/C*, *trxA4*, *trxA5*, *trxB*, *trxB2,* and *trxB3* in wt and Δ*adpA* when induced by 300 µM HS_n_H. The *hrdB* transcription was used as the internal control for normalization. The expression level of each gene in wt without induction was arbitrarily set to 1, and the fold changes in expression levels in wt and Δ*adpA* without induction (green bars) and with HS_n_H induction (brown bars) were shown. All data were averages from three samples with standard deviation (error bar). *T*-tests were performed to calculate the *p*-values, and asterisks indicate statistically significant difference (* *p* < 0.05, ** *p* < 0.01).

To examine whether the expression of thioredoxins was induced by RSS, we performed HS_n_H induction experiments. The wt and Δ*adpA* strains were cultivated in liquid YBP medium for 36 h (exponential phase). 300 µM HS_n_H was added, and the cultivation was continued for 1 h. Untreated cultures were used as the controls. Cells were harvested for RNA extraction and RT-qPCR analysis. In wt, the expression levels of *trxA* (*sco3889*, Gene ID: 1099325), *trxB2* (*sco6834*, Gene ID: 1102273), and *trxB3* (*sco7298*, Gene ID: 1102736) were significantly increased after HS_n_H treatment; the expression levels of *trxA2* (*sco5438*, Gene ID: 1100878), *trxA3/C* (*sco0885*, Gene ID: 1096308), *trxA5* (*sco1084*, Gene ID: 1096507), and *trxB* (*sco3890*, Gene ID: 1099326) were less significantly increased, while the expression level of *trxA4* (*sco5419*, Gene ID: 1100859) was not significantly increased (Figure 3). In Δ*adpA*, for except *trxB3* (*sco7298*), other thioredoxins did not show significantly increased expression after HS_n_H treatment. For each thioredoxin, the expression level in wt was significantly higher than that in Δ*adpA* after HS_n_H treatment (Figure 3). These results indicate that RSS-containing compounds induce the expression of thioredoxins, which requires the presence of AdpA.

### 3.4. Overexpressing Thioredoxins Promoted H_2_S Release

**Figure 4:**
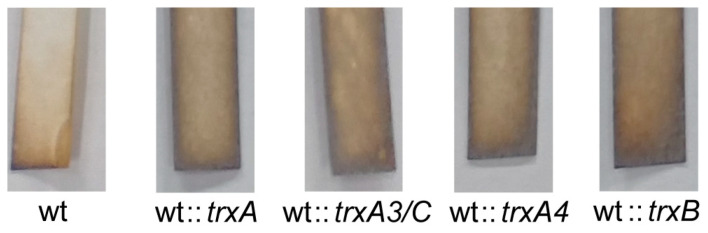
The effect of overexpression *trx* genes in *S. coelicolor* M145 on H_2_S production. H_2_S production by *S. coelicolor* M145 wt, wt::*trxA*, wt::*trxA3/C*, wt::*trxA4*, and wt::*trxB* in YBP medium between 24 h and 48 h culture was detected in the gas phase with lead–acetate paper. The experiment was repeated at least three times.

To test whether thioredoxins were involved in the process of S^0^ reduction to H_2_S, we overexpressed *trxA*, *trxA3*, *trxA4*, or *trxB* genes in *S. coelicolor* M145. Lead–acetate test strips [Pb(Ac)_2_] were used to detected the production of H_2_S. The test strip was attached to the inside of the shaker containing 100 mL YBP medium and replaced with a new test strip after 24 h. The amount of H_2_S produced was compared by the color of the strip (more H_2_S leads to darker color). The overexpression thioredoxins led to increased H_2_S release (Figure 4), suggesting that these thioredoxins catalyze the reduction of RSS to H_2_S.

### 3.5. AdpA Directly Regulated the Transcription of Trx Genes

**Figure 5:**
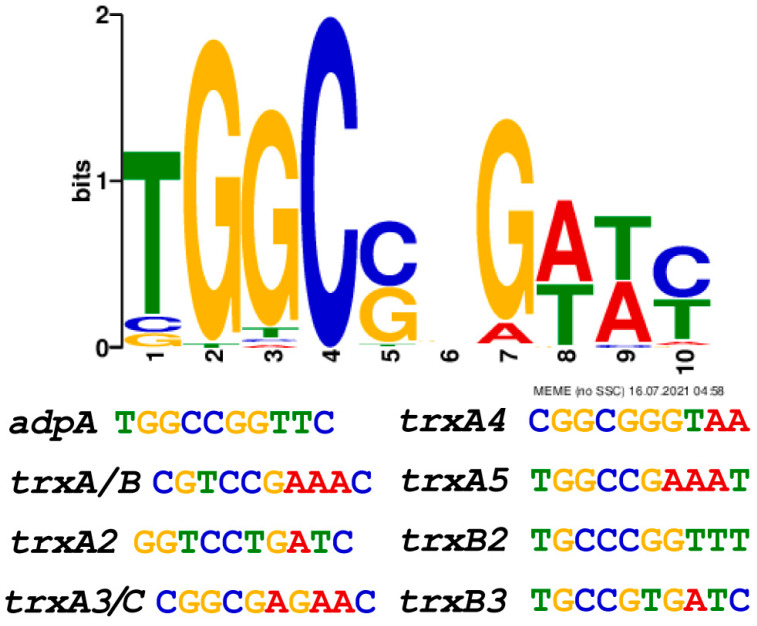
The consensus sequence for AdpA binding predicted by MEME analysis. The sequence contains nine relative conserved and one non-conserved (the sixth) nucleotides.

**Figure 6:**
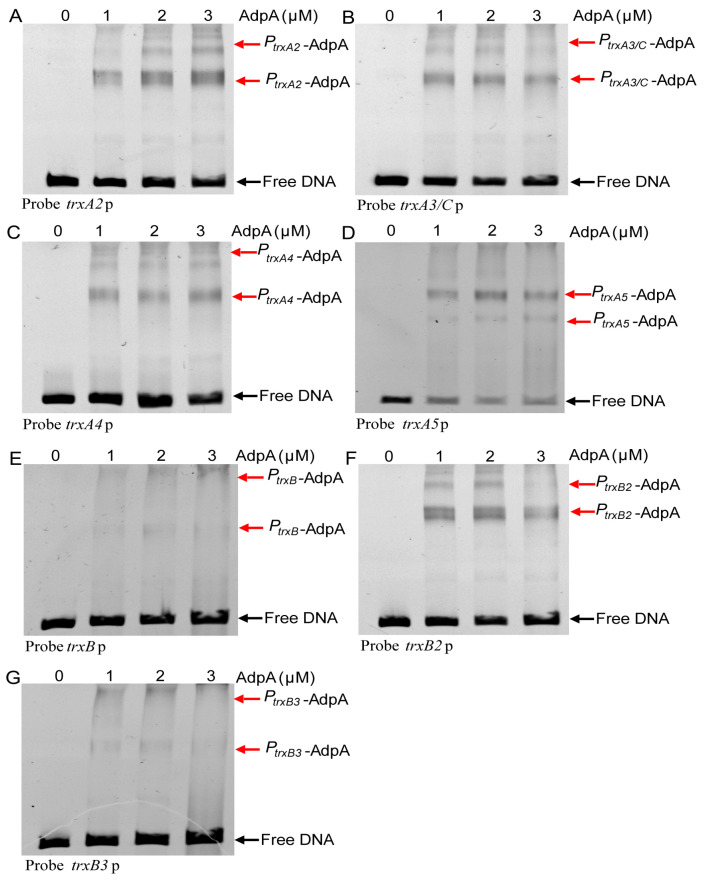
EMSA analysis His-tagged AdpA with the upstream promoter sequence probes of *trx* genes. 20 nM DNA probe was incubated with different amounts of His-tagged AdpA (0, 1, 2, 3 µM). Black arrow indicates the free DNA probe and red arrow indicates the AdpA-DNA complex. (**A**) A 299-bp DNA fragment containing the promoter and partial ORF sequence of *trxA2* (*sco5438*) was synthesized as the probe. (**B**) A 311-bp DNA fragment containing the promoter and partial ORF sequence of *trxA3/C* (*sco0885*) was synthesized and used as the probe. (**C**) A 335-bp DNA fragment containing the promoter and partial ORF sequence of *trxA4* (*sco5419*) was synthesized and used as the probe. (**D**) A 155-bp DNA fragment containing the promoter and partial ORF sequence of *trxA5* (*sco1084*) was synthesized and used as the probe. (**E**) A 334-bp DNA fragment containing the promoter and partial ORF sequence of *trxB* (*sco3890*) was synthesized and used as the probe. The *trxA* and *trxB* were co-transcribed, *trxB* the first gene of the operon. (**F**) A 285-bp DNA fragment containing the promoter and partial ORF sequence of *trxB2* (*sco6834*) was synthesized and used as the probe. The probe is promoter, (**G**) A 277-bp DNA fragment containing the promoter and partial ORF sequence of *trxB3* (*sco7298*) was synthesized and used as the probe.

We previously reported that RSS releases the transcriptional self-inhibition on AdpA expression, thereby increasing AdpA levels [33]. We suspected that over-expressed AdpA can activate expression of thioredoxins. We analyzed the promoters of thioredoxin genes using the MEME software (http://meme-suite.org/tools/meme, accessed on 25 January 2023). The binding consensus sequence of AdpA is 5′-TGGCSNGWWY-3′ (S: G or C; W: A or T; Y: T or C; N: any nucleotide) [46,47]. This consensus sequence was found in all seven *trx* promoters (*trxA* and *trxB* are co-transcribed) (Figure 5). For experimental confirmation, we used synthesized promoter probes and purified AdpA to perform EMSA and observed that AdpA bound to all seven promoter probes (Figure 6A–G). These findings suggest that AdpA directly regulates the transcription of these *trx* genes.

We also mixed AdpA and HS_n_H at a molar ratio 1:50 (AdpA/HS_n_H); the binding of AdpA to seven *trx* promoters showed no obvious change (data not shown). These results suggested that increasing AdpA expression is the main mechanism for RSS to activate the expression of thioredoxins.

## 4. Discussion

**Figure 7:**
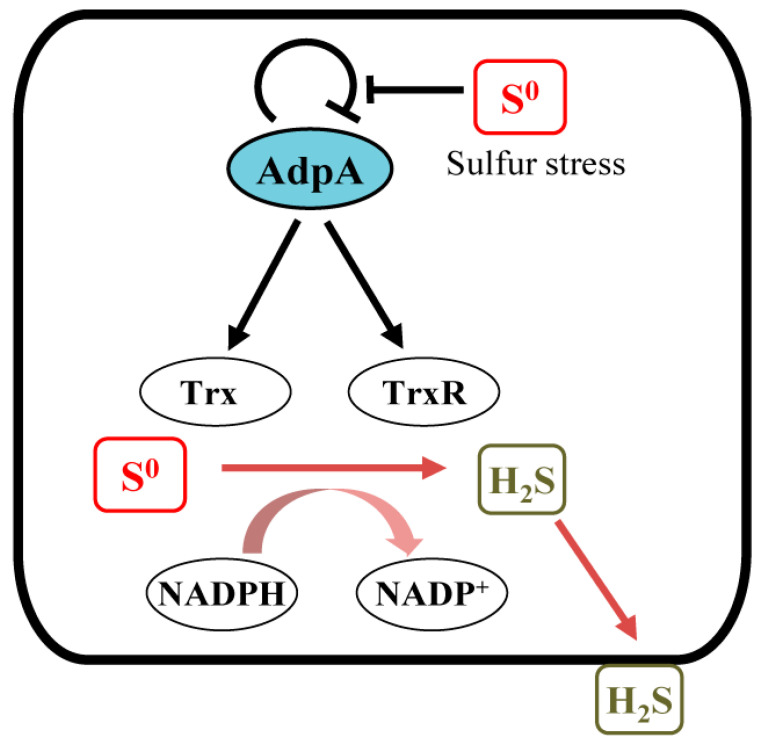
The proposed model of AdpA regulates the removal of excess intracellular RSS via thioredoxin systems in *Streptomyces coelicolor*.

Living organisms are always exposed to different environmental stresses and have developed specialized strategy to cope with them. Several transcription factors dealing with RSS stress have been identified. They sense intra- and extracellular RSS and maintain the intracellular RSS homeostasis. For instance, CstR negatively regulates the transcription of H_2_S oxidation genes in *Staphylococcus aureus* [48]. SqrR is responsible for the regulation of 45% sulfide-responsive genes in *Rhodobacter capsulatus* [49]. FisR transcriptionally activates the expression of H_2_S oxidation genes in *Cupriavidus pinatubonensis* [50] and *Acinetobacter baumannii* [51]. In addition to these RSS specific sensors, a few global or multifunctional regulators also have RSS sensing functions, such as MgrA in *Staphylococcus aureus* [52], MexR in *Pseudomonas aeruginosa* [53], OxyR in *Escherichia coli* [54], and AdpA in *S. coelicolor* [33]. In a previous study, we demonstrated that RSS can release the self-inhibition of AdpA expression via modifying its Cys^62^ residue, thereby affecting secondary metabolites production and morphological differentiation [33]. In this study, we demonstrated that AdpA is also responsible for maintaining intracellular RSS homeostasis. Deleting *adpA* makes *S. coelicolor* more sensitive to exogenous RSS stresses, suggesting that AdpA can activate expression of *trx* genes, whose products (thioredoxins) can reduce RSS to H_2_S, which evaporates into the gas phaseto relieve the RSS stress (Figure 7). AdpA is global transcriptional regulator widely present in *Streptomyces* spp. AdpA binds to more than hundreds of operator regions, and these sequences are less conserved. The crystal structure of the AdpA-DBD-DNA complex and mutation analysis of AdpA-DBD revealed its unique DNA recognition mode, with only two arginine residues directly recognizing the conserved site, explaining its strict recognition of G and C at position 2 and 4, respectively, as well as its tolerance recognition to other positions in the consensus sequence [55]. Although AdpA has been reported to regulate many important physiological processes [56,57,58,59], this study is the first report of its role in the maintenance of intracellular RSS homeostasis. Our finding revealed a new role that AdpA may commonly play in *Streptomyces* spp.

We previously demonstrated that the H_2_O_2_-response regulator OxyR senses intracellular RSS and activates the expression of catalase (KatG), glutaredoxin A (GrxA), and thioredoxin C (TrxC) in *E. coli.* These enzymes remove excess RSS via either oxidation or reduction [54]. AdpA also senses RSS and activates thioredoxins expression. These studies suggest that thioredoxins, conserved in many bacteria, constitute a general mechanism used by bacteria to maintain intracellular RSS homeostasis.

However, it is noteworthy that during the late period of cultivation, the intracellular RSS content of Δ*adpA* sharply decreased (Figure 2). This phenomenon suggests that AdpA might not be the only regulator controlling thioredoxins expression or that the removel of RSS is due to other factors other than thioredoxins. Further studies are thus required to elucidate the cause of this abrupt decrease of the RSS content in the Δ*adpA* mutant.

Notably, actinorhodin (ACT) is highly correlated with oxidative response. ACT is a pH-responsive benzoisochromanequinone produced by a type II polyketide synthase-based pathway. Its quinone groups participate in the redox-cycling reactions. The reduced form may react with oxygen to produce superoxide anions, which in turn generate reactive oxygen species (ROS) [60]. This may be one of the reasons why ACT has bactericidal properties. The oxidized ACT can act as an oxidative biocatalyst to react with L-ascorbic acid and L-cysteine to produce hydrogen peroxide (H_2_O_2_) [61]. This observation is consistent with the view that ACT as a redox antibiotic to perform its sterilizing function. ACT biosynthesis can be triggered by common oxidative stress such as nitric oxide (NO), RSS, and ROS has been recently reported [35,62,63,64,65]. These results suggest that ACT is both an oxidant and an antioxidant. ACT can be used as an endogenous signaling molecule to induce the expression of the redox-sensitive regulator SoxR and its regulon in *S. coelicolor* [66,67]. SoxR directly regulates the thioredoxins to promote resistance of thiol-oxidative stress in *Streptomyces avermitilis* [68]. We speculate that SoxR is not the only regulatory factor involved in antioxidant defense and ACT biosynthesis. In this study, AdpA not only directly regulates antibiotic production, but also regulates the oxidative stress resistance of thioredoxins. Deletion of *adpA* resulted in low expression of thioredoxins and accumulation of oxidant RSS, and AdpA is essential for the production of antioxidant ACT, aggravating the stress sensitivity of the cells. It was further confirmed that antibiotic production was a survival strategy to cope with the hostile environment in *Streptomyces*, however, the mechanism needs to be further explored.

## 5. Conclusions

Sulfane–sulfur compounds containing active sulfur atoms with a valence state of 0 or −1 are common components of cells. Sulfane sulfur is the inducer of numerous regulatory factors and participates in many physiological functions and metabolic pathways. Given the oxidizing and reducing properties of sulfane sulfur, it can be both oxidant and antioxidant. A high concentration of sulfane sulfur causes oxidative stress response and damages cells. Organisms develop different strategies to remove excess RSS in cells and maintain redox balance, but more studies are required to comprehensively understand the mechanisms. In this study, we demonstrated that the global regulator AdpA was critical for eliminating excessive RSS in *S. coelicolor*. The sensitivity of *adpA* deletion mutant to sulfur stress was significantly increased and the endogenous RSS accumulated in the mutant. *Streptomyces* has multiple pathways and enzymes to protect itself from oxidative stress, of which the thioredoxin systems are commonly involved. Overexpression of these thioredoxin systems resulted in more H_2_S release in *S. coelicolor*. The results showed that thioredoxin systems affected sulfur metabolism and reduced intracellular RSS. In addition, AdpA significantly activated the expression of thioredoxins and thioredoxin reductases to deal with sulfur stress. Overall, we found that AdpA has the regulatory function to promote sulfane sulfur reduction and maintain redox balance. This discovery further expands the pathway of antioxidant regulation in *Streptomyces*.

## Data Availability

The data are contained within the article and supplementary materials.

## Supplementary Materials

The following supporting information can be downloaded at: https://www.mdpi.com/article/10.3390/antiox12020312/s1, Table S1. Strains and plasmids used in this study. Table S2. Primers sequence (5′→3′) used in this study.
