# Supplementary material for: The Pleiotropic Regulator AdpA Regulates the Removal of Excessive Sulfane Sulfur in Streptomyces coelicolor
Source: Antioxidants (Basel). 2023 Jan 29;12(2):312. doi: 10.3390/antiox12020312 (PMC9952706; doi:10.3390/antiox12020312)
Supplement: Supplementary file 1 [file antioxidants-12-00312-s001.zip › Table S1 Strains and plasmids used in this study.pdf]

Table S1. Strains and plasmids used in this study.

Strain or plasmid	Description	Resource
***S. coelicolor* strains**		
M145	Wild type strain that produces multiple antibiotics, spc1$^-$ and spc2$^-$	Lab stock
Δ*adpA*	M145 strain with disruption of *adpA* gene, Apr^R	[1]
Δ*adpA:: adpA*	Δ*adpA* complemented with pComadpA, Apr^R, Hyg^R	[1]
Δ*adpA::* pMS82	Δ*adpA* complemented with pMS82 vector, Apr^R, Hyg^R	[1]
wt:: *trxA4*	wt complemented with pCom trxA4, Hyg^R	This study
wt:: *trxA*	wt complemented with pCom trxA, Hyg^R	This study
wt:: *trxB*	wt complemented with pCom trxB, Hyg^R	This study
wt:: *trxA3*	wt complemented with pCom trxA3, Hyg^R	This study
***E. coli* strains**		
DH5*α*	Cloning strain	Lab stock
BL21 (DE3)	Host used for protein expression	Lab stock
ET12567(pUZ8002)	Strain used for conjugation, with plasmid pUZ8002, Km^R,Cm^R	Lab stock
BL21 (DE3)AdpA	Protein expression host with pET-AdpA, Amp^R	This study
**Plasmids**		
pJTU1278	The shuttle vector for gene mutation, Amp^R	Lab stock
pJTU-*adpA*	pJTU1278 derivative for *adpA* disruption, Apr^R, Amp^R	[1]
pIJ773	Template for apramycin resistance gene, Apr^R	Lab stock
pET-15b	Expression vector for proteins, Amp^R	Lab stock
pEX-AdpA	*adpA* expression vector, Amp^R	[1]
pMS82	Integrative vector for gene complementation, Hyg^R	Lab stock
pMS82-*trxA4*	pMS82 derivative for *trxA4* (*sco5419*) gene complementation, Hyg^R	This study
pMS82-*trxA*	pMS82 derivative for *trxA* (*sco3889*) gene complementation, Hyg^R	This study
pMS82-*trxB*	pMS82 derivative for *trxB* (*sco3890*) gene complementation, Hyg^R	This study
pMS82-*trxA3*	pMS82 derivative for *trxA3* (*sco0885*) gene complementation, Hyg^R	This study

1. Lu, T.; Wu, X.; Cao, Q.; Xia, Y.; Xun, L.; Liu, H. Sulfane sulfur posttranslationally modifies the global regulator AdpA to influence actinorhodin production and morphological differentiation of *Streptomyces coelicolor*. *mBio* **2022**, *13*, e0386221.
