# Supplementary material for: The Pleiotropic Regulator AdpA Regulates the Removal of Excessive Sulfane Sulfur in Streptomyces coelicolor
Source: Antioxidants (Basel). 2023 Jan 29;12(2):312. doi: 10.3390/antiox12020312 (PMC9952706; doi:10.3390/antiox12020312)
Supplement: Supplementary file 1 [file antioxidants-12-00312-s001.zip › Table S2 list of primers used in this study.pdf]

Table S2. Primers sequence (5′→3′) used in this study.

Primers	Sequence (5'-3')	Function
hrdB-realtime-f	CGCCGAGTCCGTCTCTGTCA	Real-time PCR for reference gene
hrdB-realtime-r	GCTCTGCGGCACTGACCATC	(Gene ID: 1101262)
trxA(sco3889)-realtime-f	GACTGACGACTCCTTCGAGC	Real-time PCR for *trxA*(*sco3889*)
trxA(sco3889)-realtime-r	GATGGACATGACGCCGTACT	(Gene ID: 1099325)
trxA3/C(sco0885)-realtime-f	GAACTTCGACCAGACGGTCA	Real-time PCR for *trxA3*/*trxC* (*sco0885*) (Gene ID: 1096308)
trxA3/C(sco0885)-realtime-r	GCGTCGGGATCGAACTGAT	
trxA4(sco5419)-realtime-f	CCCGGTCGTCATCGACTTC	Real-time PCR for *trxA4*(*sco5419*)
trxA4(sco5419)-realtime-r	GAGGAAGCGCCCGTTGTATT	(Gene ID: 1100859)
trxB(sco3890)-realtime-f	CGAGCTCATGGACAACATGC	Real-time PCR for *trxB*(*sco3890*)
trxB(sco3890)-realtime-r	GTCGGTGACGGTCTTGATCT	(Gene ID: 1099326)
trxB3(sco7298)-realtime-f	TGAACGGCATCGAGTTCCTC	Real-time PCR for *trxB3*(*sco7298*)
trxB3(sco7298)-realtime-r	TGAGCAGGTAGTGGTCCAGA	(Gene ID: 1102736)
trxB2(sco6834)-realtime-f	CCACGACTGAGGTCGAGAAC	Real-time PCR for *trxB2*(*sco6834*)
trxB2(sco6834)-realtime-r	TCTTGACGTCACCGGTCAGA	(Gene ID: 1102273)
trxA5(sco1084)-realtime-f	ACAGTTCTCCAGTGCCTTCTG	Real-time PCR for *trxA5*(*sco1084*)
trxA5(sco1084)-realtime-r	GTCGATCTCGACGTGGGC	(Gene ID: 1096507)
trxA2(sco5438)-realtime-f	GAGTTCACCGCCGACTGG	Real-time PCR for *trxA2*(*sco5438*)
trxA2(sco5438)-realtime-r	ATCGACAGCACCTTGTACGC	(Gene ID: 1100878)
adpA-exp-f	GTGCCGCGCGGCAGCCATATGAGC CACGACTCCACCGCCGCGC	AdpA recombinant expression (Gene ID: 1098226)
adpA-exp-r	GTTAGCAGCCGGATCCTCGAGTCA CGGCGCGCTGCGCTGGCCCGGG	
trxA5(sco1084)EMSA-f	TACCAGCCTTGGTGCCCGTG	155bp fragment for EMSA
trxA5(sco1084)EMSA-r	TCATGGGCGCGCGTCCTGCG	
trxA3/C (sco0885)EMSA-f	TTGCTGGGCATGCACGCCCAGTA	311bp fragment for EMSA
trxA3/C (sco0885)EMSA-r	CTTGGTGAGTTCCACGGTGCTGGT	
trxB(sco3890)EMSA-f	GATCACGTTTCGGACGTCGCTCA	334bp fragment for EMSA
trxB(sco3890)EMSA-r	CTACAAGGGCAGGGAAGCCTTGCT	
trxA4(sco5419)EMSA-f	GCTGCTCTGCATCCCCCGACCTTA	335bp fragment for EMSA
trxA4(sco5419)EMSA-r	AGGTCGACGACCCCGCTCATGGA	
trxA2(sco5438)EMSA-f	GCGGCAGATCAGGACCCTCCAGGA	299bp fragment for EMSA
trxA2(sco5438)EMSA-r	AAGTCCGCGTCCGTCACCTCGG	
trxB2(sco6834)EMSA-f	CGCACGTCCTTGGCGGTGCTCATG A	285bp fragment for EMSA
trxB2(sco6834)EMSA-r	CCGTCGAGGCCATGGCCGAGGT	
trxB3(sco7298)EMSA-f	TGTCTCCCCGTCGGCCTGTGCCATA	277bp fragment for EMSA
trxB3(sco7298)EMSA-r	TCTCCTCCGAACCGTCCGAGCCTG C	
pMS82-kp-trxA F	TCTAAGTAAGGAGACATCTAGTGG CCGGCACCCTGAAGCATGTGA	Complementary *trxA* by *kasOp** promoter

pMS82-kp-trxA R	TGAAAAACGCTCACTGGTACCTCA GTCGGCGATGAAGTCCTCGA	
pMS82-kp-trxA3 F	TCTAAGTAAGGAGACATCTAATGA CCAGCACCGTGGAACTCACCA	Complementary *trxA3* by *kasOp** promoter
pMS82-kp-trxA3 R	TGAAAAACGCTCACTGGTACCCTA CTGGCCTTCCTGGCCTTCCT	
pMS82-kp-trxA4 F	TCTAAGTAAGGAGACATCTAATGA GCGGGGTCGTCGACCTCGCCGC	Complementary *trxA4* by *kasOp** promoter
pMS82-kp-trxA4 R	TGAAAAACGCTCACTGGTACCCTA GAACAGGGCGCGGGCGAGCG	
pMS82-kp-trxB F	TCTAAGTAAGGAGACATCTAGTGA GCGACGTCCGAAACGTGATCA	Complementary *trxB* by *kasOp** promoter
pMS82-kp-trxB R	TGAAAAACGCTCACTGGTACCTCA GACGGCGGTCTTCTCGGGCT	
